# RIVA – a phase IIa study of rituximab and varlilumab in relapsed or refractory B-cell malignancies: study protocol for a randomized controlled trial

**DOI:** 10.1186/s13063-018-2996-6

**Published:** 2018-11-09

**Authors:** Sean H. Lim, Kim M. Linton, Graham P. Collins, Joke Dhondt, Joshua Caddy, Liz Rossiter, Karan Vadher, Keira Fines, Laura E. Rogers, Diana Fernando, Louise Stanton, Andrew J. Davies, Peter W. M. Johnson, Gareth Griffiths

**Affiliations:** 1 Antibody and Vaccine Group, Centre for Cancer Immunology, University of Southampton, Southampton, UK; 2 Division of Cancer Sciences, School of Medical Sciences, Faculty of Biology, Medicine and Health, University of Manchester, Manchester Cancer Research Centre, Southampton, UK; 3 Department of Haematology, Oxford University Hospitals NHS Foundation Trust, Oxford, UK; 4 Southampton Clinical Trials Unit, Centre for Cancer Immunology, University of Southampton, Southampton, UK; 5 ECMC Southampton, University of Southampton, Southampton, UK; 6 CRUK Centre Southampton, University of Southampton, Southampton, UK

**Keywords:** lymphoma, B-cell malignancy, varlilumab, rituximab, CD20, CD27, monoclonal antibody, immunotherapy, phase IIa, randomised trial

## Abstract

**Background:**

Over 12,000 new cases of B-cell malignancies are diagnosed in the UK each year, with diffuse large B-cell lymphoma (DLBCL) and follicular lymphoma (FL) being the most common subtypes. Standard frontline therapy consists of immunochemotherapy with a CD20 monoclonal antibody (mAb), such as rituximab, delivered in combination with multi-agent chemotherapy. Despite being considered a treatable and potentially curable cancer, approximately 30% of DLBCL cases will relapse after frontline therapy. Advanced stage FL is incurable and typically has a relapsing and remitting course with a frequent need for re-treatment*.* Based on supportive preclinical data, we hypothesised that the addition of varlilumab (an anti-CD27 mAb) to rituximab (an anti-CD20 mAb) can improve the rate, depth and duration of the response of rituximab monotherapy in patients with relapsed or refractory B-cell malignancies.

**Methods/design:**

Combination treatment of varlilumab plus rituximab, in two different dosing regimens, is being tested in the RIVA trial. RIVA is a two-stage open-label randomised phase IIa design in up to 40 patients with low- or high-grade relapsed or refractory CD20^+^ B-cell lymphoma. The study is open to recruitment in the UK. Enrolled patients are randomised 1:1 to two different experimental varlilumab to rituximab combinations.

The primary objective is to determine the safety and tolerability of the combination and the anti-tumour activity (response) in relapsed or refractory B-cell malignancies. Secondary objectives will include an evaluation of the duration of the response and overall survival. Tertiary translational objectives include assessment of B-cell depletion, changes in immune effector cell populations, expression of CD27 as a biomarker of response and pharmacokinetic properties. Analyses will not be powered for formal statistical comparisons between treatment arms.

**Discussion:**

RIVA will determine whether the combination of rituximab and varlilumab in relapsed or refractory B-cell malignancies is active and safe prior to future phase II/III trials.

**Trial registration:**

EudraCT, 2017–000302-37. Registered on 16 January 2017. ISRCTN, ISRCTN15025004. Registered on 16 August 2017.

**Electronic supplementary material:**

The online version of this article (10.1186/s13063-018-2996-6) contains supplementary material, which is available to authorized users.

## Background

Over 12,000 new cases of B-cell malignancies are diagnosed in the United Kingdom each year [1]. B-cell malignancies can be divided broadly into high-grade (e.g. diffuse large B-cell lymphoma [DLBCL]) or low-grade diseases (e.g. follicular lymphoma [FL] and chronic lymphocytic leukaemia/small lymphocytic lymphoma [CLL/SLL]). DLBCL, CLL/SLL and FL are the three most common subtypes, accounting for 80% of B-cell malignancies. High-grade lymphomas are potentially curable whereas low-grade lymphomas have a relapsing remitting course [2] and are incurable. Standard frontline therapy for most B-cell malignancies consists of immunochemotherapy with rituximab, a CD20 monoclonal antibody (mAb), delivered in combination with multi-agent chemotherapy, which has been shown to increase responses by up to 20% in FL and DLBCL [3–6]. It is also employed as a single agent in some indolent lymphomas [7].

DLBCL is a treatable and potentially curable cancer but approximately 30% of patients relapse after frontline therapy [8]. Salvage platinum-based chemotherapy followed by high-dose chemotherapy and an autologous stem cell transplant is offered to responsive patients who are fit for intensive treatment, but only ~ 30% of patients achieve durable remission [9]. There is no established standard for patients with relapsed DLBCL who are unfit for intensive therapy. Thus, the majority of patients with relapsed DLBCL will eventually succumb to the disease. Whilst the low-grade B-cell malignancies can often be re-treated, successive remissions become increasingly shorter in duration and usually require different therapeutic approaches. Thus, there is a clear clinical need for novel therapeutic agents in B-cell lymphoma to increase the depth and duration of response.

Rituximab is a direct tumour-targeting mAb binding the CD20 molecule on the surface of normal and malignant B cells. CD20 mAbs destroy tumour cells mainly through antibody-dependent cellular cytotoxicity and/or phagocytosis (ADCC/ADCP) (reviewed in [10, 11]). Here, the mAb engages immune effector cells, such as macrophages, through the Fc:Fcγ receptor interaction with subsequent cytolysis or phagocytosis of the target cell. There is now good evidence in preclinical models that monocytes and macrophages are the key effector cells in mediating ADCC/ADCP with CD20 mAb [12–14].

A further class of mAbs that has garnered considerable interest recently is the immunomodulatory mAbs, which can be further subdivided into immunostimulatory mAbs and immune checkpoint inhibitors. Unlike tumour-targeting mAbs, these mAbs bind to host immune cells and mediate enhanced tumour-specific T-cell responses by augmenting immune cell expansion, survival and/or function (reviewed in [15]).

Varlilumab (1F5, CDX-1127) is a recombinant and fully human IgG1 kappa and first-in-class agonistic mAb that binds with high affinity to the human tumour necrosis factor receptor (TNFR) superfamily member CD27 [16]*.* CD27 is constitutively present on all subsets of T cells [17], on a subset of natural killer (NK) cells [18] and on memory B cells [19]. Engagement of CD27 by its ligand, CD70, or an agonistic mAb leads to recruitment of TNFR-associated factor (TRAF) proteins to the CD27 cytoplasmic tail [20, 21]. Subsequent activation of canonical and non-canonical nuclear factor-kB (NF-kB) and c-Jun-N-terminal kinase (JNK)-signalling pathways follows to elicit cellular responses [22]. Activation of CD27 is critical to CD8 T-cell priming [23–26] and contributes substantially to the secondary CD8 T-cell response by enhancing memory CD8 T-cell expansion, survival and cytolytic activity [27–30]*.*

The agonistic activity of varlilumab has been demonstrated through in vitro assays where it has been shown to enhance T-cell proliferation in the presence of T-cell receptor stimulation [16] and in vivo in human CD27 transgenic mice [31]. The antibody is also capable of mediating ADCC, and regulatory T-cell depletion was observed in cynomolgus macaques [31] and in a phase I study in humans [32].

In the phase 1 dose escalation safety and pharmacokinetic study of varlilumab, 90 participants with relapsed or refractory haematological malignancies and solid tumours were enrolled (NCT01460134) [32, 33]. Varlilumab was well tolerated with the majority of adverse events (AEs) reported being mild to moderate in severity. Grade 3 treatment-related AEs reported were hyponatraemia, anorexia, raised alkaline phosphatase, lymphopenia and hypertension, and grade 4 asthma was reported on one occasion. One case of dose-limiting toxicity (DLT) was reported in which a patient with ovarian cancer experienced transient grade 3 hyponatraemia. Significant and durable responses were observed in two participants (renal cell carcinoma and Hodgkin’s lymphoma). Thirteen additional participants experienced stable disease. In summary, varlilumab was well tolerated and demonstrated agonistic activity expected of a CD27 mAb. There is clinical evidence of single-agent clinical activity in this heavily pre-treated population of patients with progressive and metastatic disease after other immunomodulatory mAbs have failed.

Our recent preclinical data support the combination of a CD27 agonist with a tumour-targeting mAb [34]. Using a variety of immunocompetent, syngeneic mouse tumour models (BCL_1_ lymphoma, A31 lymphoma Eμ-TCL1 B-cell leukaemia and B16 melanoma), and a panel of immunomodulatory mAbs (e.g. CTLA4, PD1, PDL1, 4-1BB, OX40 and GITR mAbs), we demonstrated that only anti-CD27 significantly enhances the efficacy of anti-CD20. Here, anti-CD27 stimulates T and NK cells to release IFNγ and chemokines, such as CCL3, CCL4 and CCL5, to attract and promote the ADCP capacity of myeloid cells, such as macrophages. Importantly, these findings were validated in BCL_1_-bearing hCD27 transgenic mice where varlilumab was employed. Thus, we hypothesised that the addition of varlilumab to rituximab therapy can improve the anti-tumour efficacy of rituximab and hence the rate, depth and duration of the response in patients with CD20-expressing B-cell lymphoma.

## Methods/design

The RIVA trial is a multicentre, randomised phase IIa trial evaluating whether varlilumab in combination with rituximab is active and safe in participants with relapsed or refractory CD20^+^ B-cell malignancies.

### Objectives

The primary objective is to determine the safety, tolerability and the anti-tumour activity of combined rituximab and varlilumab therapy in relapsed or refractory CD20^+^ B-cell malignancies. Secondary objectives include the evaluation of the duration of response (progression-free survival) and overall survival over a follow-up period of 1 year.

Tertiary translational objectives include the assessment of:

1. The level of B-cell depletion in the peripheral blood and, where relevant, the tumour site following therapy. B-cell depletion and intratumoural B-cell levels will be assessed using flow cytometry, in pre- and post-treatment samples.
2. The proportion of immune effector cell populations in the peripheral blood and, where relevant, the tumour site following therapy. Peripheral blood and intratumoural immune cell subset levels (CD4 and CD8 T-cell subsets, NK cells, neutrophil, monocyte and macrophage levels) will be measured by flow cytometry, in pre- and post-treatment samples.
3. The expression of CD27 as a biomarker of response in combined rituximab and varlilumab therapy. CD27 expression levels on CD8 T-cells, effector CD4 T-cells, regulatory CD4 T-cells, NK cells and B cells in pre-treatment peripheral blood and intratumoural material will be measured by flow cytometry.
4. Whether co-administration of rituximab and varlilumab alters their pharmacokinetic properties. Pharmacokinetic levels in peripheral blood from six participants in arm A will be measured.

### Treatment arms

The treatment arms are:

- Arm A
  - Cycle 1
    - Day 1: Rituximab 375 mg/m^2^ intravenously (IV)
    - Day 2: Varlilumab 3 mg/kg IV
- Arm B
  - Cycle 1
    - Day 1: Rituximab 375 mg/m^2^ IV
    - Day 8: Varlilumab 3 mg/kg IV
- In both arms A and B:
  - Cycles 2 to 6
    - Day 1: Rituximab 375 mg/m^2^ IV
    - Cycles 3 and 5
    - Day 2: Varlilumab 3 mg/kg IV

### Study design

**Fig. 1:**
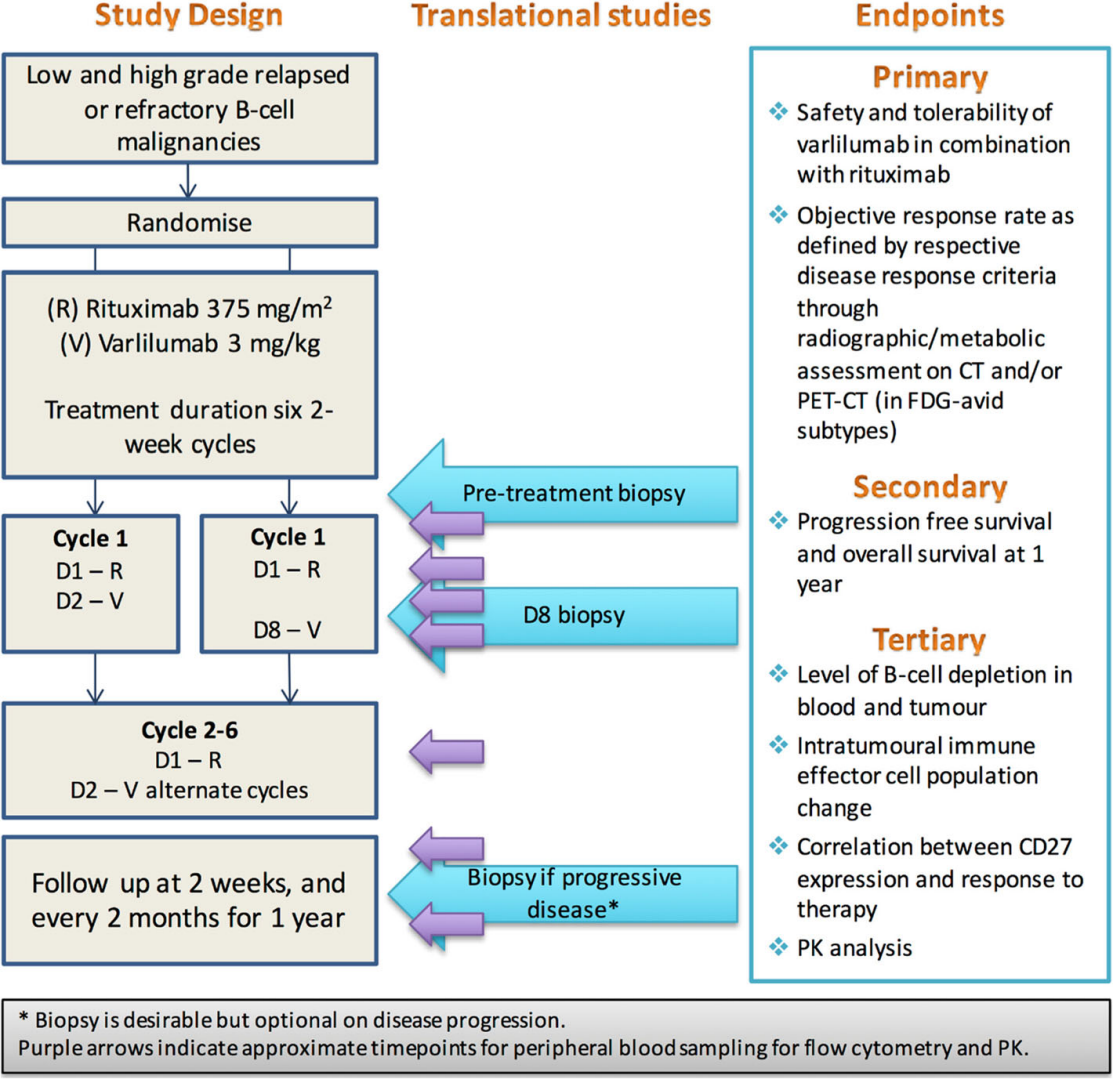
Trial flow chart. D1 day 1, D2 day 2, D8 day 8, R rituximab, V varlilumab

This is a multicentre, randomised, phase IIa trial of two experimental arms. The trial will enrol participants with relapsed or refractory CD20^+^ B-cell malignancies of low-grade and high-grade B-cell lymphoma subtypes (Fig. 1). 

RIVA will be run in four centres in the UK.

Patients will be randomised by clinic staff at each site between arms A and B, using a web-based system in a 1:1 ratio using minimisation (with a random element) and stratified for disease subtype and centre. Treatment allocation will be unblinded. There will be 10 patients per treatment arm for each of the low and high-grade subtypes, giving a total of 40 patients.

The cycle duration is 2 weeks and a maximum of six cycles will be delivered. Pre-medication with oral paracetamol 1 g and IV chlorphenamine 10 mg will be administered as standard at least 30 min prior to the start of each infusion. Hydrocortisone 100 mg IV or dexamethasone 8 mg IV will be administered prior to infusions if there has been an infusion reaction to previous rituximab therapy.

### Endpoints

For the co-primary endpoints, activity (i.e. response) will be measured according to the Lugano Revised Response Criteria for Malignant Lymphoma 2 weeks after the end of trial treatment [35, 36]. At baseline, at each treatment cycle and at each follow-up visit, safety will be assessed by determining the causality of each AE and grading to the Common Terminology Criteria for Adverse Events (CTCAE), version 4.03, which was developed by theNational Cancer Institute. Treatment compliance will be assessed using data collected in electronic case report forms during the treatment period. The secondary endpoints are progression-free survival and overall survival, measured as time-to-event (time from randomisation to event, i.e. progression or any death). Patients who do not experience the event will be censored at their last follow-up visit.

The study will be conducted in two stages as follows.

#### Stage 1 – safety

During the safety phase, six participants (three from each arm and from any subtype) will be treated. The number of DLTs experienced by these participants in each arm after having completed the first cycle will dictate whether we proceed to the second stage of the trial.

**Fig. 2:**
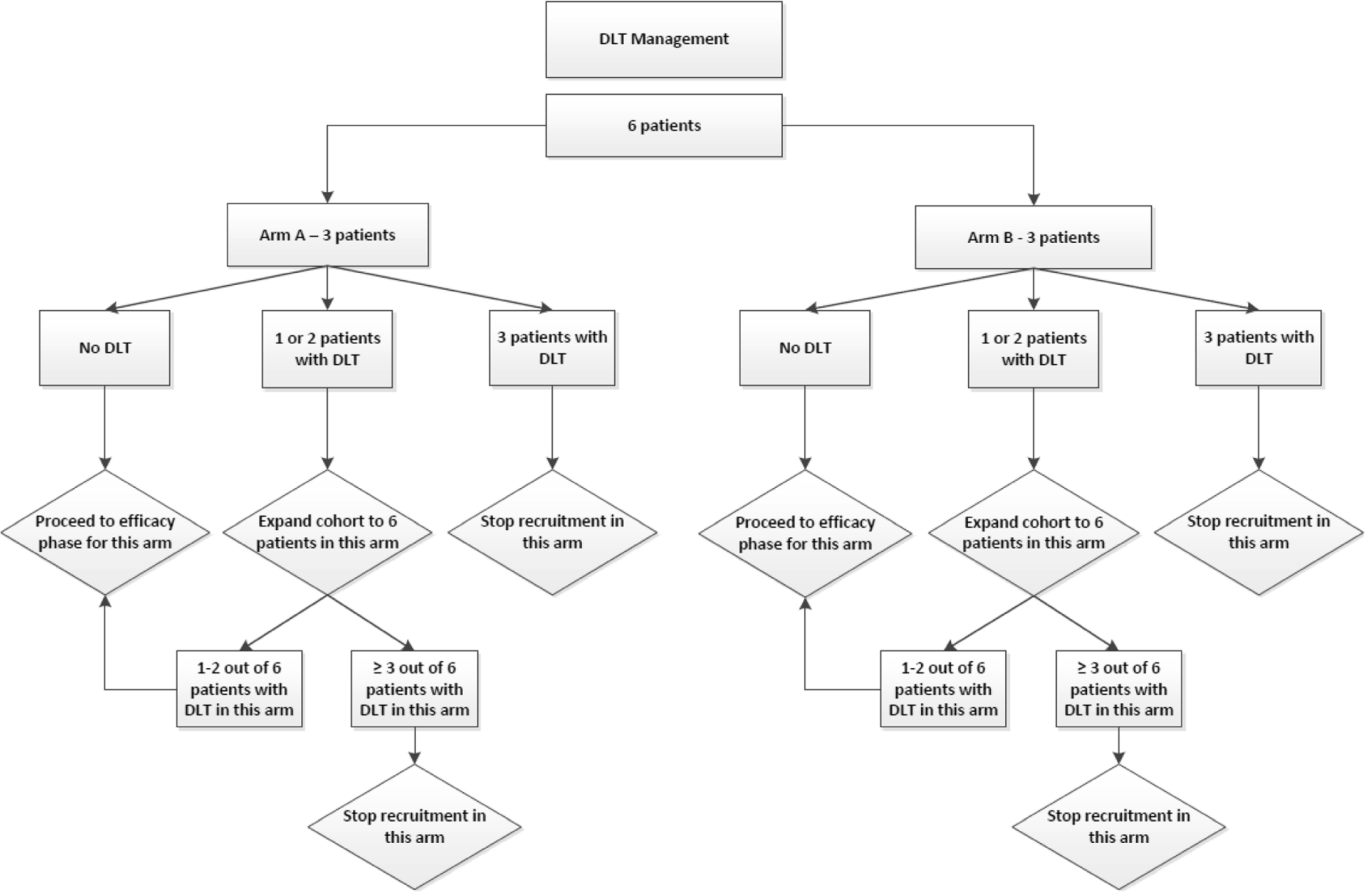
Evaluation of safety stage. DLT dose-limiting toxicity

The options are as follows (Fig. 2):

1. In each arm, if none of the three participants experience DLT, then we will proceed to stage 2.
2. In each arm, if one or two out of three participants experience DLT, then we will expand the cohort to three more participants.
  - If at most one or two out of the six participants experience DLT, we will proceed to stage 2.
  - If three or more out of the six participants experience DLT, recruitment for that arm will be stopped.
3. If all three of these participants experience DLT, recruitment for that arm will be stopped.

#### Definition of DLT

DLT, measured using CTCAE, version 4.03, is defined as a highly probable or probable treatment-related AE that occurs between the first dose of varlilumab and day 1 of the second cycle of treatment. Specifically, the relevant AEs are:

- Grade 4 neutropenia >7 days’ duration despite treatment with granulocyte-colony stimulating factor (G-CSF)
- Febrile neutropenia (fever of unknown origin without clinically or microbiologically documented infection) with grade 3 or 4 neutropenia (absolute neutrophil count, ANC < 1.0 × 10^9^/L and fever > 38.5 °C)
- Thrombocytopenia grade 4 for > 5 days or associated with active bleeding despite support
- Grade 3 or 4 cytokine release syndrome or infusion-related reactions despite pre-medication
- Grade 3 or 4 non-haematological toxicity, including grade 3 and 4 biochemical AEs
- Event with a fatal outcome

AEs that are not relevant for DLT include:

- Grade 3 nausea or grade 3 or 4 vomiting in participants who have not received optimal treatment with anti-emetics
- Grade 3/4 diarrhoea in participants who have not received optimal treatment with antidiarrhoeals
- Lymphopenia

Initially, the first patient will be entered into the trial, providing there are no serious or unexplained safety issues during cycle 1, as determined by the safety review committee (SRC). The dosing of subsequent participants will continue as they are identified. Should toxicity findings of concern occur, the SRC may choose to stagger the start of dosing for subsequent participants and/or cohorts. Recruitment was paused after the first participant was registered. At the first SRC meeting, the members of the committee agreed that recruitment need not be paused while waiting for the second SRC meeting to take place.

 Depending on the tolerability of the combination, the number of patients recruited into stage 1 of the trial will range from 6 to 12.

#### Stage 2 – activity, safety and feasibility

The objective of stage 2 is to obtain further information on the safety of the intervention in a larger sample, information on activity (response rate overall and per lymphoma subtype) and the feasibility of administrating rituximab and varlilumab together. During stage 2, recruitment will continue until there is a total of 10 participants per arm and per grade (low/high). Including those recruited in stage 1, there will be a total of 20 participants per grade, and 40 participants in the trial in total. The main purpose for having two experimental treatment arms is to provide a comparator for the translational endpoints, i.e. to assess whether the differences observed are due to the addition of varlilumab to rituximab.

In both stages, no dose reductions are allowed for rituximab or varlilumab, but treatment may be interrupted or discontinued, or the infusion rate may be changed, at the discretion of the investigator for severe infusion or allergic reactions, or other toxicities. Guidelines on the management of infusion-related reactions are provided in Additional file 1.

A new cycle of treatment can be administered to a participant if the following conditions are met:

- ANC ≥1.0 × 10^9^/L (or not lower than ANC at screening). G-CSF support is permitted at the investigator’s discretion.
- Platelets ≥75 × 10^9^/L (or not lower than platelet count at screening). This platelet count must not be supported by platelet transfusions.
- Severity of non-haematological toxicity reduced to grade 1 or less (or grade 2 or less at the investigator’s discretion, if not considered a safety risk). Further guidance on the management of non-haematological toxicity is available on request.

The administration of rituximab and varlilumab must be delayed by 1 week if these conditions are not met. If toxicities have not improved to the limits described above, treatment may be delayed by a further week. Initiation of the next cycle can be delayed by a maximum of 3 weeks. Thereafter, if the toxicity has not improved to the limits above, treatment according to the trial protocol will be permanently discontinued for that participant.

To validate our preclinical observations, pre- and post-treatment biopsies will be performed to monitor the level of B-cell depletion and the composition of other immune cell subsets. A comparator arm with rituximab alone would have been ideal, but this would not be an effective treatment option in this population. The only difference between arms A and B is the variation in the timing of the administration of varlilumab in cycle 1. This enables the collection of a post-treatment biopsy from patients who have received rituximab only and those who have received both rituximab and varlilumab, thus allowing a biological comparison of the effects of varlilumab alone. If these two experimental arms are shown to be active and safe, then a future randomised phase trial comparing the combination against a control arm will be undertaken.

### Ethical and regulatory aspects

RIVA has received ethical approval from the South Central Oxford A Ethics Committee (17/SC/0317) and has been approved by the UK Medicines and Healthcare Products Regulatory Agency (MHRA). Southampton Clinical Trials Unit (SCTU), which receives core funding from Cancer Research UK (CRUK) and is registered with the UK Clinical Research Collaboration, is coordinating the trial. A list of recruiting sites can be obtained from the SCTU. University Hospital Southampton NHS Foundation Trust is the sponsor for the trial [37]. The RIVA trial management group includes oncologists, patient and public involvement representatives, and SCTU staff involved in the day-to-day running of the trial. An independent trial steering committee has been established. The RIVA SRC comprises the principal investigators, an independent oncology clinician, statisticians and SCTU staff. The SRC reviews and assesses safety and tolerability data to make protocol-defined decisions regarding trial progress during stage 1, to advise the trial management group and trial steering committee on the conduct of stage 1, and to make a recommendation on whether to continue into stage 2. In addition, a data monitoring and ethics committee comprising two clinicians and a statistician experienced in this research area will be constituted on the launch of stage 2, to monitor trial progress and safety. Charters for these groups are available via riva@soton.ac.uk.

The SCTU has undertaken a risk assessment for the RIVA trial, which includes the requirements for monitoring (both central and on-site). The SCTU undertakes a number of internal audits of its own systems and processes annually and is routinely audited by both its sponsor and the independent MHRA every 2–3 years.

The trial is registered on the trial portfolio managed by UK National Institute for Health Research (NIHR), which means there are research nurses based in UK cancer hospitals who can help in screening potential patients to identify those eligible for the trial.

### Study participants

The RIVA trial is currently recruiting patients with relapsed or refractory CD20^+^ B-cell lymphoma for both stage 1 and stage 2

#### Inclusion criteria

To be eligible, patients must meet all of the following inclusion criteria:

1. Relapsed or refractory CD20^+^ B-cell lymphoma:
  High-grade subgroup: DLBCL, FL grade 3b or transformed FL
  Low-grade subgroup: All low-grade CD20^+^ B-cell lymphoma subtypes excluding CLL/SLL (e.g. FL grade 1, 2 or 3a, mantle cell lymphoma, marginal zone lymphoma and lymphoplasmacytic lymphoma)
2. Disease must be recurrent or treatment refractory, and the patient must have received at least one line of treatment. Rituximab-refractory participants are eligible for entry into the study as long as the tumour expresses CD20.
3. At least one measurable lesion in a computed tomography scan (defined as >1.5 cm in one axis) that is also easily accessible for biopsy
4. Histological confirmation of relapse with 12 months of treatment
5. 16 years of age or older
6. Haematological and biochemical indices with the ranges shown below:
  Haemoglobin (Hb) ≥90 g/L (red cell support is permissible)
  ANC ≥1.0 × 10^9^/L (or ≥0.5 × 10^9^/L if bone marrow involvement). G-CSF support is not permissible at screening.
  Platelet count ≥75 × 10^9^/L (or ≥ 30 × 10^9^/L if bone marrow involvement)
  Serum bilirubin ≤1.5 × upper limit of normal unless raised due to Gilbert’s syndrome, in which case up to 3 × upper limit of normal is permissible
  Alanine aminotransferase and aspartate aminotransferase ≤2.5 × upper limit of normal unless raised due to hepatic involvement
  Calculated creatinine clearance (Cockroft–Gault formula) ≥30 ml/min (uncorrected value)
7. Ability to understand the purpose and risks of the study and provide written informed consent
8. Willing and able to participate in all required evaluations and procedures in the study protocol
9. Willing to participate in appropriate pregnancy prevention measures:
  Women with childbearing potential who have a negative serum or urine pregnancy test during screening (within 14 days prior to the start of trial treatment) and who agree to use one highly effective form of contraception combined with an effective form of contraception from the first administration of all study drugs throughout the trial and for 12 months after the last dose of all study drugs.
  Male participants with partners of childbearing potential who agree to take measures not to father children by using one form of highly effective contraception from the first administration of all study drugs, throughout the trial and for 12 months after the last dose of all study drugs. Male subjects must also refrain from donating sperm during this period.
  Men with pregnant or lactating partners must use a barrier method of contraception (for example, condoms plus spermicidal gel) to prevent exposure to the foetus or neonate.
10. Life expectancy ≥12 weeks
11. ECOG performance status 0–2

Contraception that is considered highly effective includes: oral, injected or implanted progesterone-only hormonal contraception (with inhibition of ovulation); oral, intravaginal or transdermal combined (oestrogen and progesterone containing) hormonal contraception (with inhibition of ovulation); an intra-uterine device; an intra-uterine hormone-releasing system; bilateral tubal occlusion; vasectomy or abstinence.

Contraceptive methods considered to be effective include progesterone-only oral hormonal contraception, where inhibition of ovulation is not the primary mode of action, condoms, caps, and diaphragms or sponges with spermicidal gel.

#### Exclusion criteria

Patients who meet any of the following criteria will be excluded:

1. Patients with known central nervous system involvement by lymphoma that is not in remission
2. History of other malignancy within the last 2 years except for:
  Non-invasive malignancies, such as adequately treated ductal carcinoma in situ of the breast, non-melanoma skin cancer or lentigo maligna, cervical carcinoma in situ and urothelial papillary non-invasive carcinoma or carcinoma in situ
  Prostate intraepithelial neoplasia without evidence of prostate cancer
3. Receiving (or within a month of) chemotherapy, immunotherapy or treatment with immunosuppressive agents. This includes any systemic steroids at a dose exceeding 10 mg prednisolone (or other steroid equivalent) within 2 weeks prior to the first dose of varlilumab
4. Significant concurrent, uncontrolled medical condition that, in the opinion of the investigator, contraindicates participation in this study
5. Active and documented autoimmune disease (including, but not limited to, inflammatory bowel disease, coeliac disease, haemolytic anaemia or immune thrombocytopenic purpura) prior to the first dose of varlilumab
6. Active infection requiring systemic therapy
7. Women who are pregnant or lactating
8. Serological positivity for hepatitis B (HBV) or C, or known HIV infection. As per the standard of care, the results of hepatitis serology should be known prior to commencement of immunochemotherapy
  Positive test results for chronic HBV infection (defined as positive HBsAg serology and positive HBcAb). Occult or prior HBV infection (defined as negative HBsAg and positive HBcAb). Participants who have protective titres of hepatitis B surface antibody (HBsAb) after vaccination will be eligible
  Positive test results for hepatitis C (antibody serology testing)
9. Previous recipient of an allogeneic bone marrow transplant at any time
10. Autologous bone marrow transplant within 100 days of first dosing
11. Systemic radiation therapy within 4 weeks or prior focal radiotherapy within 2 weeks prior to first dosing
12. Known or suspected of being unable to comply with the protocol
13. Ongoing toxic manifestations of previous treatments. Exceptions to this are alopecia or certain grade 1 toxicities that, in the opinion of the investigator, should not exclude the patient
14. Uncontrolled congestive cardiac failure, cardiac ischaemia or cardiac arrhythmia. Clinically significant cardiac disease including unstable angina, acute myocardial infarction within 6 months prior to registration or congestive heart failure (NYHA III–IV)
15. Known hypersensitivity to rituximab (grade 3 or higher) or murine proteins, or any other excipients used in the formulation of rituximab

#### Withdrawal criteria

Participants are free to withdraw consent from the study at any time without providing a reason. A participant may also withdraw from receiving study treatment but may not wish to withdraw from the trial. In this instance, the participant will be encouraged to attend follow-up visits in accordance with the trial schedule. Should any participant become pregnant during the trial, study treatment will be discontinued.

### Study procedure

#### Recruitment and consent

Patients are approached within a hospital setting and screened for eligibility by research staff to ensure all inclusion and exclusion criteria are met. Informed consent to enter the trial is obtained from a patient by a clinician only after a full explanation has been given, a patient information sheet has been provided and time has been allowed for consideration. Patients may provide written consent up to 28 days prior to randomisation. Patients are also asked to consent to provision of tumour and blood samples for use in laboratory studies, including genetic analysis, and to consent to their data being shared anonymously to support other research in the future (see Additional file 2).

#### Baseline visit

**Fig. 3:**
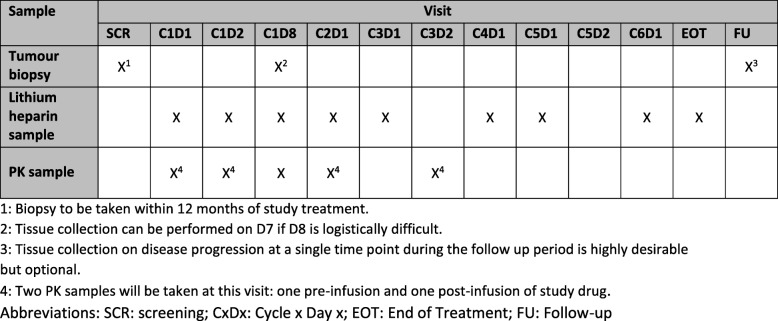
Overview of translational samples. C*x*D*x*: Cycle *x* Day *x*, EOT end of trial, FU follow-up, PK pharmacokinetics, SCR screening

**Fig. 4:**
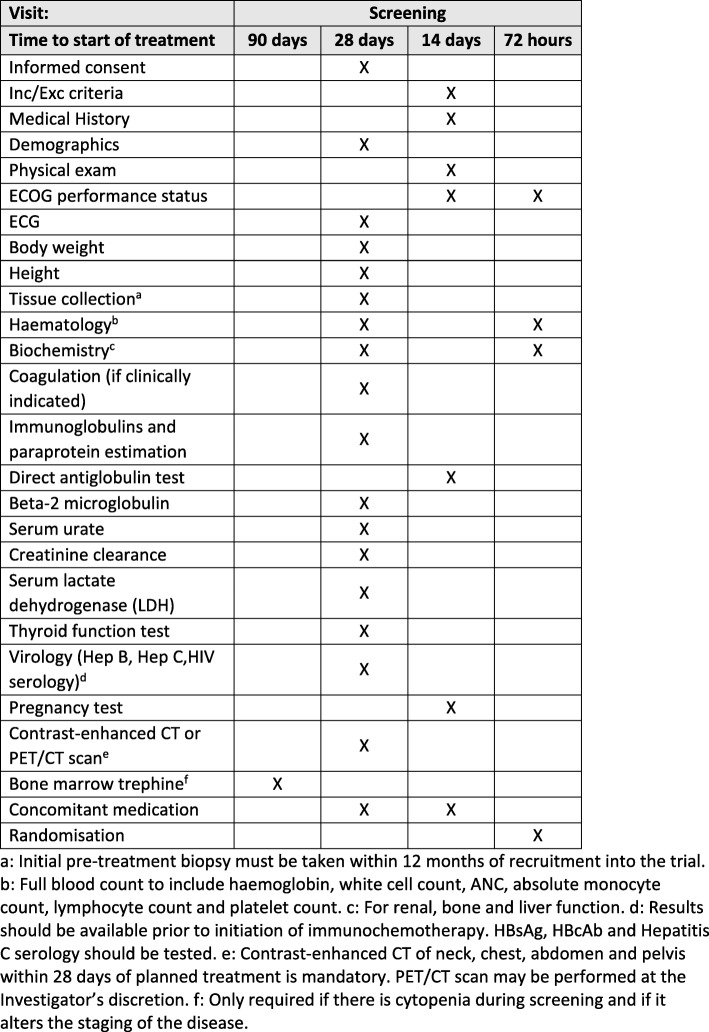
Screening and randomisation. CT computed tomography, ECG electrocardiogram, Exc exclusion, Hep hepatitis, HIV human immunodeficiency virus, Inc inclusion, PET positron emission tomography

Following informed consent, assessments including a physical examination; full blood count; direct antiglobulin test; serum biochemistry, including renal, liver and bone profiles; creatinine clearance; immunoglobulins and paraprotein estimation; beta-2 microglobulin; serum lactate dehydrogenase; thyroid function test; serology (hepatitis B or C and HIV) and an electrocardiogram are completed within 28 days prior to treatment commencing, with disease evaluation being undertaken through contrast-enhanced computed tomography or PET-CT (positron emission tomography–computed tomography) in accordance with local policy and routine practice for the relevant disease site. Concomitant medications, ECOG performance status and medical history will be recorded within 14 days of treatment. In addition, women of childbearing potential will undertake a pregnancy test within 14 days. Following registration, fresh tumour tissue will be obtained through a needle biopsy for translational work (Fig. 3). Randomisation will occur 72 h prior to treatment start and where necessary, a further full blood count, serum biochemistry and ECOG performance status will be assessed (Fig. 4).

#### Treatment and follow-up visits

**Fig. 5:**
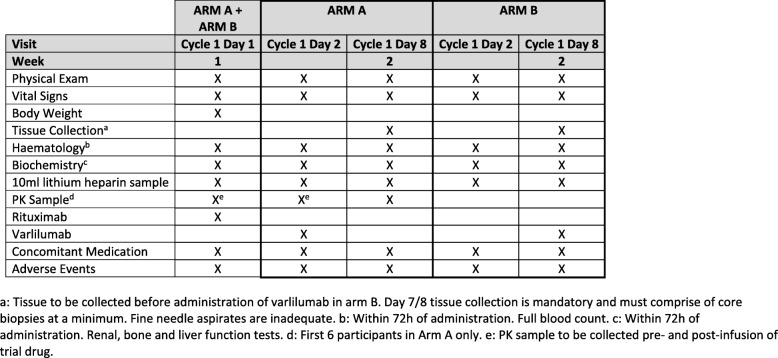
Assessments during treatment and administration of treatment (cycle 1). PK pharmacokinetics

**Fig. 6:**
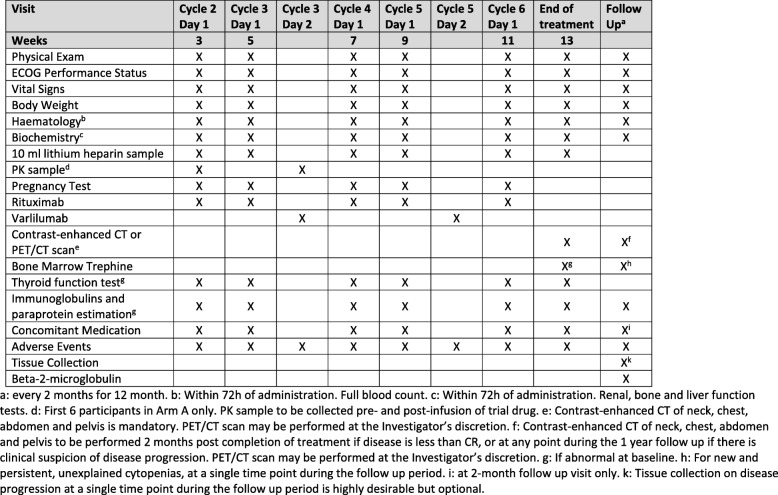
Assessments during treatment (cycles 2–6), at end of treatment and at follow-up (arms A and B), and administration of treatment. CT computed tomography, PK pharmacokinetics, PET positron emission tomography

Participants attend hospital appointments for treatment cycles with assessments as per the baseline visit plus assessments of AEs, blood samples for translational analyses and pharmacokinetics (Fig. 3) and treatment compliance. Following the treatment, participants will attend post-treatment and progression follow-up visits where data will be collected on AEs, disease status and survival status (Figs. 5 and 6). Serious adverse events (SAEs) will be reported in real time to the SCTU pharmacovigilance team throughout the study. SAEs are assessed to determine whether they are related to drug treatment and whether they were unexpected. SAEs are subsequently reported to both Celldex, who are one of the manufacturers of varlilumab and who provide it for this trial, and the appropriate UK regulatory bodies.

### Data collection

Research staff at hospitals will complete trial electronic case report forms via a remote data collection tool (Medidata Rave). Data will be checked for missing or unusual values and checked for consistency within participants over time by SCTU trial staff. Any inconsistencies in data will be raised as data queries with the relevant site. Site staff will respond to such queries to provide an explanation or resolution of the discrepancies. Full details of the data management procedures are available in the RIVA data management plan, available on request.

### Source document verification and monitoring

The trial will be monitored and audited in accordance with SCTU procedures. In stage 1, every time a patient is registered, a monitor from the SCTU will visit the site to verify the source data. This monitoring will encompass comparing entries on the trial electronic case report forms with patients’ medical records and other supporting documents at the site and documented in the monitoring report form. Details will remain confidential, consistent with data protection regulations. Drug accountability will also be monitored throughout the trial. In stage 2, the SCTU will use a risk-based monitoring process to determine monitoring frequency and extent.

### Sample size

If fewer than 13% of the participants respond to the treatment, then this combination would be deemed insufficiently active to warrant further study. If, however, 40% or more of the participants respond to the treatment then the combination would be deemed worthy of further investigation. Using a one-stage Fleming design at α = 0.05 (one-sided) and 90% power, this would require 20 participants in each of the high- and low-grade arms (arms A and B combined). If during the safety phase one of the treatment arms is closed, then all participants will be recruited to the remaining arm so that the total sample size is 20 in each disease category. A total of 40 participants will be recruited, with 20 participants in each of the following subcategories:

1. High-grade lymphoma (DLBCL, FL grade 3b or transformed FL) (*n* = 20)
2. Low-grade lymphoma (e.g. FL grade 1, 2 or 3a, marginal zone lymphoma or mantle cell lymphoma) (*n* = 20)

The decision on whether there is sufficient activity to warrant further investigation in a future phase III will based on the following criteria:

1. Within each of the high-grade and low-grade subcategories, if six or more out of the 20 participants respond to treatment.
2. There is increased intratumoural B-cell depletion in the day 8 biopsies of participants who have received rituximab and varlilumab compared to rituximab alone.
3. There is increased activation or an increase in the absolute numbers or proportions of macrophages, monocytes or neutrophils in the day 8 tumour biopsies of participants who have received rituximab and varlilumab compared to rituximab alone.

### Statistical analysis

#### Stage 1

All patients entered into stage 1 will be accounted for. The analysis will focus on the incidence of DLT, which will be summarised by treatment arm (and within the high-and low-grade subtypes). In addition, baseline characteristics, details of dose delivery and all toxicities on the CTCAE toxicity scale (version 4.03) during treatment will be summarised by grade and dose cohort. Details of dose delivery will also be summarised.

#### Stage 2

The analysis will be conducted for the intention-to-treat population, which includes all randomised patients who have commenced study treatment. We do not expect a difference in response rates by arm, so for analyses, participants from arm A and arm B will be combined. There will be no formal statistical comparisons between groups, and any *p* values presented will be exploratory (the main purpose for having two experimental treatment arms is to provide a comparator for the translational endpoints, i.e. to assess whether the differences observed are due to the addition of varlilumab to rituximab). For the primary endpoint of response, the percentage of responders and the 90% confidence interval will be presented for both the low- and high-grade groups. For other descriptive categorical data, proportions with a 90% confidence interval will be presented and for continuous data, means and a 90% confidence interval will be presented (or median and interquartile range if appropriate). For time-to-event data, Kaplan–Meier curves and waterfall plots for the response endpoint will be presented. A statistical analysis plan will be developed for the interim analysis and the final analysis.

### Interim analysis

The SRC will review the data during stage 1 to determine safety before progressing to stage 2. A data monitoring and ethics committee will monitor the trial during stage 2. Safety, activity and treatment compliance analyses will be planned and agreed with the data monitoring and ethics committee in advance. It is anticipated that all patients who have been randomised will be included in these analyses. All analyses will be carried out using STATA version 15 and SAS version 9 or later.

### Adverse event reporting

Data on AEs will be collected at treatment and follow-up visits. SAEs will be reported until 180 days after the last administration of trial drugs. The trial also has a UK regulatory compliant real-time SAE reporting process to identify serious adverse reactions and suspected unexpected serious adverse reactions that could lead to the suspension or cessation of the trial if warranted.

### End of the trial

The end of trial is defined as when all data items have been collected from all patients.

### Trial status

This clinical trial was registered on 16 January 2017 on EudraCT (2017–000302-37), and on 16 August 2017 on ISRCTN (ISRCTN15025004). Recruitment opened on 23 November 2017 and is expected to be completed in May 2019. The first patient was registered on 29 January 2018. This study protocol was written in accordance with Standard Protocol Items: Recommendations for Interventional Trials (SPIRIT). A SPIRIT checklist is provided in Additional file 3. The current protocol is version 3, dated 5 July 2017. Protocol amendments that have been approved by the research ethics committee and the MHRA will be communicated to sites via email. Updated trial documentation will be available centrally via the trial website. Trial registrations will be amended where relevant with explanations for these changes.

## Discussion

The outcome of this trial will provide evidence for whether the combination of rituximab and varlilumab in relapsed or refractory B-cell malignancies is active and safe prior to future phase II/III trials. Confirmation of enhanced B-cell depletion and increased infiltration of myeloid cells with the addition of varlilumab to rituximab, as observed in our preclinical data, will provide a proof of principle that CD27 agonism does indeed enhance the efficacy of tumour-targeting mAbs. Anti-CD27 may also enhance the efficacy of other ADCP-dependent tumour-targeting mAbs in other cancers, such as anti-EGFR in head and neck squamous cell carcinoma and anti-GD2 in neuroblastoma. Other potential prognostic and predictive markers will later be explored through RNA sequencing. Validation and verification of any identified biomarkers will need to be performed in retrospective studies or further prospective trials. By repeated tumour sampling, the project aims to characterise changes in the tumour and its microenvironment through protein and mRNA expression. The ability to correlate these changes with clinical responses is key to understanding how we can further improve mAb therapy in resistant cases.

Our results will be disseminated to patients and clinical teams through peer-reviewed journal articles authored by the members of the trial management group and presented at international conferences.

## Additional files

Additional file 1: Detailed information on RIVA dose modification and management of infusion-related reactions. This guidance is to be used if a patient experiences peri-infusional reactions. (PDF 88 kb)

Additional file 2: Copy of the consent form given to all study participants. (PDF 178 kb)

Additional file 3: Standard Protocol Items: Recommendations for Interventional Trials (SPIRIT): a checklist for a set of scientific, ethical and administrative elements recommended to be listed in a protocol [38]. (PDF 58 kb)

## Abbreviations

ADCC: Antibody-dependent cell-mediated cytotoxicity
ADCP: Antibody-dependent cellular phagocytosis
AE: Adverse event
ANC: Absolute neutrophil count
CLL: Chronic lymphocytic leukaemia
CRUK: Cancer Research UK
CTCAE: Common terminology criteria for adverse events
DLBCL: Diffuse large B-cell lymphoma
DLT: Dose-limiting toxicity
ECOG: Eastern Cooperative Oncology Group
EGFR: Epidermal growth factor receptor
FL: Follicular lymphoma
G-CSF: Granulocyte-colony stimulating factor
Hb: Haemoglobin
HBV: Hepatitis B virus
HIV: Human immunodeficiency virus
IRAS: Integrated Research Application System
IV: Intravenously
mAb: Monoclonal antibody
MHRA: Medicines and Health Care Product Regulatory Agency
NHS: National Health Service
NIHR: National Institute for Health Research
NK: Natural killer
NYHA: New York Heart Association
PET-CT: Positron emission tomography–computed tomography
SAE: Serious adverse event
SCTU: Southampton Clinical Trials Unit
SLL: Small lymphocytic lymphoma
SPIRIT: Standard Protocol Items: Recommendations for Interventional Trials
SRC: Safety review committee
TNFR: Tumour necrosis factor receptor
