# Supplementary material for: RIVA – a phase IIa study of rituximab and varlilumab in relapsed or refractory B-cell malignancies: study protocol for a randomized controlled trial
Source: Trials. 2018 Nov 9;19:619. doi: 10.1186/s13063-018-2996-6 (PMC6230275; doi:10.1186/s13063-018-2996-6)
Supplement: Supplementary file 1 — Detailed information on RIVA dose modification and management of infusion-related reactions. This guidance is to be used if a patient experiences peri-infusional reactions. (PDF 88 kb) [file 13063_2018_2996_MOESM1_ESM.pdf]

Additional File 1: Management of Infusion Related Reaction (IRR)

Recommendations for the management of peri-infusional reactions are provided below and may be modified based on local treatment standards and guidelines, as appropriate. Infusion related reactions should be graded according to NCI-CTCAE (Version 4.0) guidelines.

**Severity of IRR (per NCI CTCAE Grade)**	**Dose Modification**
**Grade 1 symptoms** (mild transient reaction; infusion interruption not indicated; intervention not indicated)	Remain at bedside and monitor patient until recovery from symptoms.
**Grade 2 symptoms** (infusion interruption indicated but responds promptly to symptomatic treatment [e.g., antihistamines, nonsteroidal anti-inflammatory drugs, opioids, corticosteroids, IV fluids]; prophylactic medications indicated for ≤ 24 hours)	Stop the varlilumab infusion, begin an IV infusion of sodium chloride 0.9%, and treat the patient with chlorphenamine 10 mg IV (or equivalent) and/or 500 to 1000 mg paracetamol/acetaminophen; remain at bedside and monitor patient until resolution of symptoms. Corticosteroid therapy may also be administered as appropriate. If the infusion is interrupted, then restart the infusion at 50% of the original infusion rate when symptoms resolve; if no further complications ensue after 30 minutes, the rate may be increased to 100% of the original infusion rate. Monitor patient closely. If symptoms recur then no further varlilumab will be administered at that visit. The amount of study drug infused must be recorded on the eCRF. Participants who experience an adverse event, including an infusion reaction of Grade 2, during the 4-6 hour observation period that does not resolve during this time should be observed for 24 hours or until the adverse event resolves with vital sign measurements every 4 hours and additional evaluations as medically indicated for the management of the adverse event.
**Grade 3 or Grade 4 symptoms**:<br>Grade 3: prolonged [i.e., not rapidly responsive to symptomatic medication and/or brief interruption of infusion]; recurrence of symptoms following initial improvement; hospitalisation indicated for other clinical sequelae [e.g., renal impairment, pulmonary infiltrates].<br>Grade 4: life-threatening consequences; urgent intervention indicated.	Immediately discontinue infusion of varlilumab. Begin an IV infusion of sodium chloride 0.9%, and treat the patient as follows: Recommend bronchodilators, epinephrine 0.2 to 1 mg of a 1:1,000 solution for subcutaneous administration or 0.1 to 0.25 mg of a 1:10,000 solution injected slowly for IV administration, and/or chlorphenamine 10 mg IV with methylprednisolone 100 mg IV (or equivalent), as needed. Patient should be monitored until the Investigator is comfortable that the symptoms will not recur. Investigators should follow their institutional guidelines for the treatment of anaphylaxis. Participants who experience an adverse event, including an infusion reaction of Grade ≥ 3, regardless of resolution, will be observed for 24 additional hours or until the adverse event resolves with vital sign measurements every 4 hours and additional evaluations as medically indicated for the management of the adverse event. In the case of late-occurring hypersensitivity symptoms (e.g., appearance of a localised or generalised pruritus within 1 week after treatment), symptomatic treatment may be given (e.g., oral antihistamine, or corticosteroids). Varlilumab will be permanently discontinued for participants with Grade 4 infusion reactions. Varlilumab should be permanently discontinued for recurrence of Grade 3 infusion reaction for participants on pre-medication.
