# Supplementary material for: RIVA – a phase IIa study of rituximab and varlilumab in relapsed or refractory B-cell malignancies: study protocol for a randomized controlled trial
Source: Trials. 2018 Nov 9;19:619. doi: 10.1186/s13063-018-2996-6 (PMC6230275; doi:10.1186/s13063-018-2996-6)
Supplement: Supplementary file 2 — Copy of the consent form given to all study participants. (PDF 178 kb) [file 13063_2018_2996_MOESM2_ESM.pdf]

(TO BE PRINTED ON LOCAL HOSPITAL HEADED PAPER)

CONSENT FORM

Study Title: A phase IIa study of Rituximab and Varlilumab in relapsed or refractory B-cell malignancies (RiVa)

REC Reference Number: 17/SC/0317

Patient ID Number: …………………………………………………
(to be obtained after registration)

Name of Researcher: …………………………………………………

	Please initial each box
1. I confirm that I have read and understand the information sheet (Version 3 dated 24-May-2018) for the above study and I fully understand what is involved in taking part in this trial. I have had the opportunity to ask questions and these have been answered satisfactorily.	INITIAL
2. I understand that my participation is voluntary and that I am free to withdraw at any time, without giving any reason, without my medical care or legal rights being affected.	INITIAL
3. I agree to the storage of my blood and tissue samples by the researchers conducting this study for use in research relating to the aims of this study. I understand that these samples will be stored in a licensed tissue bank.	INITIAL
4. I agree to give blood samples for the assessment of varlilumab levels in my blood (pharmacokinetics) as described in the patient information sheet for the above study. I give permission for these samples, and other information with my details, to be transferred to Celldex Therapeutics (USA-based company).	INITIAL
5. I understand that relevant sections of any of my medical records and data collected during the study, may be looked at by responsible individuals from the Sponsor or Sponsor delegates from Southampton Clinical Trials Unit, and from the Regulatory Authorities where it is relevant to my taking part on this research. I give permission for these individuals to have access to my records.	INITIAL

6. I consent to my anonymised trial data being used in future research by third parties involved in this research, including those both inside and outside the European Economic Area (for example the USA).	INITIAL
7. I understand that I shall not benefit financially, even if future research leads to the development of new treatments or medical tests.	INITIAL
8. I agree to my GP being informed about my participation in this research study.	INITIAL
9. I agree to use very effective contraception as detailed in the Patient Information Sheet and to refrain from donation of egg/sperm (if applicable) during the trial treatment and for 12 months after the last dose of the trial drug.	INITIAL
10. I agree to my pseudo-anonymised data being held on servers located in the EU and USA. Access to this data will be strictly controlled by Southampton Clinical Trials Unit (SCTU) and applicable Data Protection Legislation will be abided by.	INITIAL
11. I agree to take part in this study.	INITIAL

OPTIONAL

12. I agree that the blood samples, tissue samples, consent form and information collected about me will be stored on behalf of the RiVa Trial Management Group and may be used in future ethically approved projects. I understand that some of these projects may be carried out by researchers other than the RiVa Trial Management Group.	INITIAL

Name of patient	Date	Signature

Name of person taking consent	Date	Signature

REMINDER FOR RESEARCH TEAM:

- **File original signed consent form in Investigator Site File**
- **Give one copy to the patient**
- **File one copy in the patient's medical records**
